# Vitamin D in the General Population of Young Adults with Autism in the Faroe Islands

**DOI:** 10.1007/s10803-014-2155-1

**Published:** 2014-06-14

**Authors:** Eva Kočovská, Guðrið Andorsdóttir, Pál Weihe, Jónrit Halling, Elisabeth Fernell, Tormóður Stóra, Rannvá Biskupstø, I. Carina Gillberg, Robyn Shea, Eva Billstedt, Thomas Bourgeron, Helen Minnis, Christopher Gillberg

**Affiliations:** 1 Gillberg Neuropsychiatry Centre, Institute of Neuroscience and Physiology, Sahlgrenska Academy, University of Gothenburg, Kungsgatan 12, 411 19 Göteborg, Sweden; 2 Institute of Health and Wellbeing, College of Medical, Veterinary and Life Sciences, Caledonia House, Royal Hospital for Sick Children, University of Glasgow, Glasgow, G3 8SJ UK; 3 Genetic Biobank of the Faroes, Ministry of Health, J.C. Svabosgøta 43, 100 Tórshavn, Faroe Islands; 4 Department of Occupational Medicine and Public Health, Faroese Hospital System, Sigmundargøta 5, 100 Tórshavn, Faroe Islands; 5 Research and Development Centre, Skaraborgs Hospital, 541 85 Skövde, Sweden; 6 Psychiatric Center, The National Hospital of the Faroe Islands, J.C. Svabosgøta, 100 Tórshavn, Faroe Islands; 7 Clinical Biochemistry Department, City Hospital, Sandwell and West Birmingham Hospitals, NHS Trust, Dudley Road, Birmingham, B18 7QH UK; 8 Human Genetics and Cognitive Functions Unit, Institute Pasteur, Paris, France; 9 CNRS URA 2182 Genes, Synapses and Cognition, Institut Pasteur, Paris, France; 10 Human Genetics and Cognitive Functions, Sorbonne Paris Cité, University Paris Diderot, Paris, France

**Keywords:** Autism, ASD, Vitamin D, Calcitriol, Total population, Faroe Islands

## Abstract

Vitamin D deficiency has been proposed as a possible risk factor for developing autism spectrum disorder (ASD). 25-Hydroxyvitamin D_3_ (25(OH)D_3_) levels were examined in a cross-sectional population-based study in the Faroe Islands. The case group consisting of a total population cohort of 40 individuals with ASD (aged 15–24 years) had significantly lower 25(OH)D_3_ than their 62 typically-developing siblings and their 77 parents, and also significantly lower than 40 healthy age and gender matched comparisons. There was a trend for males having lower 25(OH)D_3_ than females. Effects of age, month/season of birth, IQ, various subcategories of ASD and Autism Diagnostic Observation Schedule score were also investigated, however, no association was found. The very low 25(OH)D_3_ in the ASD group suggests some underlying pathogenic mechanism.

## Introduction

Autism spectrum disorders (ASDs) are a heterogeneous group of complex, biologically based neurodevelopmental disorders. There are several known risk factors including a variety of mutated and variant genes, advanced paternal age, exposure to toxins and medications in early development, prematurity, and birth complications (Kolevzon et al. 2007; Gardener et al. 2009; Coleman and Gillberg 2012; Munger et al. 2012; Cannell and Grant 2013). Recently, maternal/neonatal vitamin D deficiency has been proposed as a possible environmental risk factor for ASD (Cannell and Grant 2013; Grant and Soles 2009; Kočovská et al. 2012a, b) due to its involvement in early neurodevelopment (Eyles et al. 2013), the immune system (Hayes et al. 2003), and gene regulation (Ramagopalan et al. 2010) processes.

Ergocalciferol (often called vitamin D_2_), a direct analogue of cholecalciferol (vitamin D_3_), is made from ergosterol (obtained from yeast) and has been used for food fortification and supplements. The LC–MS/MS assay in blood (used in this study, see below) can differentiate between and determine levels of both 25(OH)D_2_ and vitamin 25(OH)D_3_. The 25(OH)D_2_ level reflects vitamin D_2_ intake from supplements and the 25(OH)D_3_ level reflects the vitamin D_3_ intake from diet, supplements or sun exposure. The overall result is a sum of both circulating forms and it was this overall sum that this study has used (Feldman et al. 2011).

Indirect support for the involvement of vitamin D in ASD comes from ecological studies, according to which vitamin D levels vary with season and latitude and with the degree of skin pigmentation (Grant and Soles 2009; Dealberto 2011). The prevalence of ASD has been suggested to be raised at higher latitudes and in children of migrant mothers with darker skin (Grant and Soles 2009; Fernell et al. 2010; Dealberto 2011).

The end product of vitamin D metabolism is calcitriol (1,25-dihydroxyvitamin D_3_ or 1,25(OH)_2_D_3_) that has now been recognized inter alia as a neuroactive hormone that signals via nuclear receptors (Eyles et al. 2005, 2013). It has been shown to be required for normal brain homeostasis and brain development (Garcion et al. 2002). The last 15 years have witnessed great advances in explaining the biochemical mechanisms of the diverse actions of calcitriol in the brain, especially its role in early neurodevelopment and in degenerative processes: (1) cell differentiation and axonal growth; (2) stimulation of neurotrophic factor expression (e.g., cytokines); (3) regulation of calcium signalling directly in the brain; (4) modulation of the production of the brain-derived reactive oxygen species; (5) stimulation of glutathione (a potent anti-oxidant, involved in DNA synthesis and repair) and thereby down-regulating excitotoxicity (Eyles et al. 2005, 2013; Garcion et al. 2002). Outcomes of many of these mechanisms during neurodevelopment might be relevant in a number of Early Symptomatic Syndromes Eliciting Neurodevelopmental Clinical Examinations (ESSENCE) (Gillberg 2010)—conditions that are now being linked with deficits of this vitamin/hormone, including ASD (Eyles et al. 2013).

To date, there have only been six clinical studies measuring vitamin D levels of individuals with ASD. These are: Humble et al. (2010) (without a comparison group); Molloy et al. (2010) (with a problematic comparison group); Meguid et al. (2010); Mostafa and AL-Ayadhi (2012); De Souza Tostes et al. (2012) and Gong et al. (2014), four of which showed significantly lower levels of vitamin D in individuals with this diagnosis as compared to healthy comparison group (“Appendix”). Fernell et al. (2010) demonstrated extremely low vitamin D levels in mothers of Somali origin living in Sweden with a child with ASD as compared to control group of Swedish mothers.

An apparent epidemic of vitamin D deficiency is now being recognised (Holick 2007; Adams and Hewison 2010), and this prompted us to explore the vitamin D levels in a general population cohort of young individuals with ASD (aged 15–24 years) and their siblings and parents, and in a typically-developing comparison group in the Faroe Islands. We chose the Faroe Islands for this study for various reasons. The islands’ location in the North Atlantic Ocean at 62°00′N, and its maritime climate (high rainfall, strong winds, an average summer temperature of 9 °C), negatively affect the availability of the UVB radiation of sun rays necessary for vitamin D metabolism. Conversely, the Faroe Islanders have a diet rich in large oily fish containing vitamin D that could possibly, at least in part, compensate for the lack of UVB exposure, which in turn might mean that overall vitamin D status in the Faroe Islands could be adequate. In addition, many variables are unusually stable, including socioeconomic status, education, health care, familial/genetic history, and diet. This unique total population study in the Faroe Islands therefore offers an ideal environment for examining associations between vitamin D levels and ASD.

## Methods

### Study Population

Participants with ASD were recruited during a two-phase screening process of the entire Faroe Islands population (n = 47,962) in the relevant school age group (7–16 years, n = 7,689) for the study of ASD prevalence in the Faroe Islands in 2002 (Ellefsen et al. 2007) (n = 43) and 2009 (n = 24) (Kočovská et al. 2012a, b). Thus the 67 individuals diagnosed with ASD represent an entire age-cohort of individuals with ASD in the Faroe Islands and therefore the present study represents the first ever entire population sample of vitamin D levels in ASD population. This current cross-sectional population-based study involved 219 individuals, all of white European origin: 40 participants with a diagnosis of ASD (31 males/9 females), their 62 typically developing siblings (29 brothers/33 sisters), their 77 parents (40 mothers/37 fathers), and 40 healthy comparisons (28 males/12 females).

In 2008–2009, 40 of the 67 individuals with ASD from the general population—24 participants (56 %) from the 2002 screening phase cohort and 16 (67 %) from the 2008–2009 cohort—and their close family members agreed to have blood drawn for analysis of various environmental factors (informed consent was obtained either from the individual or, if younger than 18, from the parent). The 40 participants with ASD were 15–24 years old [Mean 18.9 (SD 2.9)] at the time of blood sampling, and 31 were male. The comparison group was matched as closely as possible for age [Mean 18.5 (SD 2.5)], season of birth and gender. The reasons for non-participation in blood sampling among the ASD group (n = 27) were as follows: non-participation in the follow-up study in 2009 of those with ASD first diagnosed in 2002 (n = 10), participation but not willingness to give blood sample (n = 14), and participation but practical difficulties in blood drawing (n = 3). The group of 40 with blood samples had a similar gender profile and Autism Diagnostic Observation Schedule (ADOS) (Lord et al. 1989) scores to those from whom blood samples were not obtained (77/73 % and 12.5/9.4 respectively, *p* = 0.1.

Although some degree of genetic relatedness in the Faroe Islands can be assumed—due to the small population and its genetic isolate character, the prevalence of autism in the Faroe Islands has been found to be similar to other western nations, namely 0.56 % in 2002 (Ellefsen et al. 2007) and 0.94 % in the follow up study in 2009 (Kočovská et al. 2012a, b). The fact that in the follow up screening process an additional 24 individuals were diagnosed with autism, who were originally missed, and nearly half of these were females (n = 11), supports the findings of other studies, suggesting that girls are often missed at a young age and that screening and diagnostic processes need to address this phenomenon in the future (Giarelli 2010; Kočovská et al. 2013). There is good evidence (e.g. Kopp et al. 2010) that girls with ASD are missed or misdiagnosed at early ages and that, in fact, they had the symptoms from a very early age. There is little to indicate that girls develop ASD later than boys. There were only two families with an index child with ASD with another sibling also diagnosed with ASD but these siblings were not part of our study due to the age restriction (our participants had to be born between 1985 and 1994). During the diagnostic process in 2009, several families had siblings with some ASD traits but without an ASD diagnosis. Thus, in this vitamin D study there were no multi-ASD families.

### Ethics

The study was approved by the Scientific Ethics Committee of the Faroe Islands.

### Clinical Evaluation of Individuals with ASD

Participants with ASD were clinically examined in depth, their family history was reported by their primary caregiver, and they were assessed using a variety of standardized structured and semi-structured instruments and tests: the diagnostic interview for social and communication disorders (DISCO) (Wing et al. 2002), Wechsler Intelligence Scales (Wechsler 1981, 1992) and the ADOS (Lord et al. 1989) (see below). All were clinically diagnosed according to the International Classification of Diseases, Tenth Edition (ICD-10) (World Health Organization 1993), and the Diagnostic and Statistical Manual of Mental Disorders, Fourth Edition (DSM-IV) (American Psychiatric Association 1994) criteria: childhood autism/autistic disorder; Asperger syndrome/disorder (without application of the age criterion); atypical autism/pervasive developmental disorder not otherwise specified (PDDNOS). The subcategories were collapsed into a broader ASD study group.

The DISCO-interview is an investigator-based structured and semi-structured instrument developed with a view to serving as a research and clinical interview with a collateral informant (usually one of the parents, as in the present context) for differential diagnosis within the spectrum of autism and other social communication disorders (Wing et al. 2002). The DISCO-10 was used in 2002, and the DISCO-11 in 2009.

The difference between the tenth (DISCO-10) and the eleventh (DISCO-11) versions of the DISCO is marginal. Stability of DISCO-algorithm subcategory diagnoses has been shown to be more variable than that of clinical diagnosis but still good for AD. In terms of “any ASD” diagnosis, DISCO-11 diagnoses showed excellent stability over the 7-year period from school age through early adult life (Kočovská et al. 2013).

Wechsler intelligence scales were used age-appropriately for the cognitive assessment: Wechsler Intelligence Scale for Children (WISC-III) (Wechsler 1992) in a majority of the cases in 2002 and Wechsler Adult Intelligence Scale (WAIS-R) (Wechsler 1981) in 2009.

ADOS-assessment was performed only in 2009. The ADOS is an instrument used for diagnosing and assessing autism, allowing a standardised assessment of autistic symptoms (Lord et al. 1989).

### Blood Sampling and Assay of 25(OH)D_3_

Over a period of a year during 2008–2009, all participants had their blood drawn (EDTA monovette) at Torshavn Hospital, Faroe Islands, to determine the levels of 25(OH)D_3_. More than three quarters of the families with an index child with autism (78 %) had their blood drawn in summer (Jun–Aug) and autumn (Sep–Nov). The rest of the families were blood sampled in spring (Mar–May). No ASD participants had their blood sample drawn during the winter months. Thus it can be expected that the ASD group might demonstrate a certain seasonal elevation in their levels of vitamin D. In contrast, all healthy comparisons had their blood drawn in schools during a relatively short period between February and April 2009. In some participants with ASD, the blood samples were drawn at their homes due to needle phobia and/or other behavioural/care-taking problems.

Samples were then frozen at −80 °C and stored at the Department of Biochemistry of the Biobank of Faroes. The long-term stability of 25(OH)D_3_ serum concentrations for more than 10 years has been demonstrated under similar storage conditions (Agborsangaya et al. 2010). The stored, frozen whole blood samples were thawed in early 2013 and the required amount of 0.5 mL of haemolysed full blood separated, packed, and posted to the Department of Clinical Biochemistry, City Hospital, Birmingham, UK, where the laboratory analyses were performed by using the “gold-standard” method—liquid chromatography–tandem mass spectrometry (LC–MS/MS), details of which have been described by (Schottker et al. 2012). The laboratory staff were blind to the identity and diagnostic/comparison status of the individuals. The assay is accredited by the Vitamin D External Quality Assessment Scheme (DEQAS) and the laboratory is CPA accredited (available at: http://www.deqas.org/).

By using the haematocrit (hct) of the sample, the concentrations of 25(OH)D_3_ were calculated after measurement of the haemolysed sample (Shea and Berg 2013) which enabled the use of the haemolysed whole blood samples. All samples from all participants and the entire control group were haemolysed.

We resolved to use the same reference range as (Holick et al. 2011), since this range has been used in several recently published studies of vitamin D levels in patients with autism (e.g. Humble et al. 2010; Meguid et al. 2010; Dalgård et al. 2010; De Souza Tostes et al. 2012). The Swedish reference range for 25(OH)D_3_ levels awaits revision and the cut off for deficiency and insufficiency are expected to correspond to 50 and 75 nmol/L respectively. This scale, based on the latest research, seems plausible and practical to adopt (Priemel et al. 2010; Heaney and Holick 2011). The cut off for ‘severe deficiency’ (25 nmol/L–10 ng/mL) fulfils a practical function in clinical settings for the prescribed supplementation of vitamin D.

The reference range used in this study:

**Table:** 

	nmol/L	ng/mL
Severe deficiency	<25	10
Deficiency	≥25–<50	10–20
Insufficiency	≥50–<75	20–29
Sufficiency	≥75	30

### Statistical Analyses

Statistical analysis was performed in Minitab (version 16.0) and SPSS (version 19). Continuous data are presented as ‘mean’ and ‘standard deviation’ (SD) if normally distributed or as ‘median’ and ‘inter-quartile range’ (IQR) if not normally distributed. All data on vitamin D levels for all groups were not normally distributed, therefore we used non-parametric tests. We treated the groups of individuals with ASD and their siblings and parents as related.

The comparison group was selected to match as closely as possible in terms of gender, season of birth and age to give comparable groups for the statistical analysis.

Group comparisons of normally distributed data were made using Student’s *t* test. For continuous data that were not normally distributed (~all vitamin D levels in all groups), Mann–Whitney tests and Kruskal–Wallis tests were used when performing pairwise-comparisons and several-group-comparisons respectively. A significance level of 0.05 was considered significant for all analyses.

A linear model was used to correct for season of sampling. The vitamin D levels were not normally distributed (Anderson–Darling *p* < 0.005) however, a log transformation of the vitamin D levels was (*p* = 0.525).

Chi squared test or the Mantel–Haenszel linear-by-linear association Chi squared test for trend was used to assess categorical variables. Pearson correlation and logistic regression were used for analysis of the association between vitamin 25(OH)D_3_ levels and certain background variables.

## Results

As there were several outliers with very high levels in all groups apart from siblings, all the statistical analyses were re-calculated with all outliers removed. However, we consider inclusion of the outliers important and as the results have remained unchanged and significant after exclusion of all the outliers, we present the original results including all the outliers unless specifically stated otherwise.

### 25(OH)D_3_ Levels

**Fig. 1:**
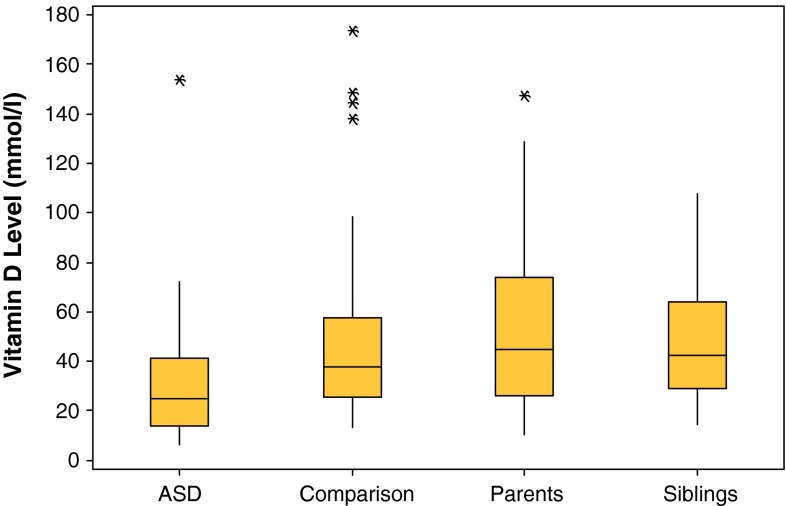
*Box plots* for 25(OH)D_3_ levels for ASD, comparison (*p* = 0.002), parent (*p* < 0.001) and sibling (*p* < 0.001) groups, (95 % CI) (*asterisk* outliers)

The ASD group had significantly lower levels of 25(OH)D_3_ [median (IQR) = 24.8 (27.5) nmol/L] compared to the healthy comparison group [median (IQR) = 37.6 (32.3) nmol/L], (95 % CI 5.0–22.5), *p* = 0.002 to their siblings [median (IQR) = 46.1 (28.3) nmol/L] (95 % CI 9.4–24.8), and parents [median (IQR) = 46.7 (36.2) nmol/L) (95 % CI 11.0–27.74], (*p* < 0.001 in both instances) (Fig. 1).

**Fig. 2:**
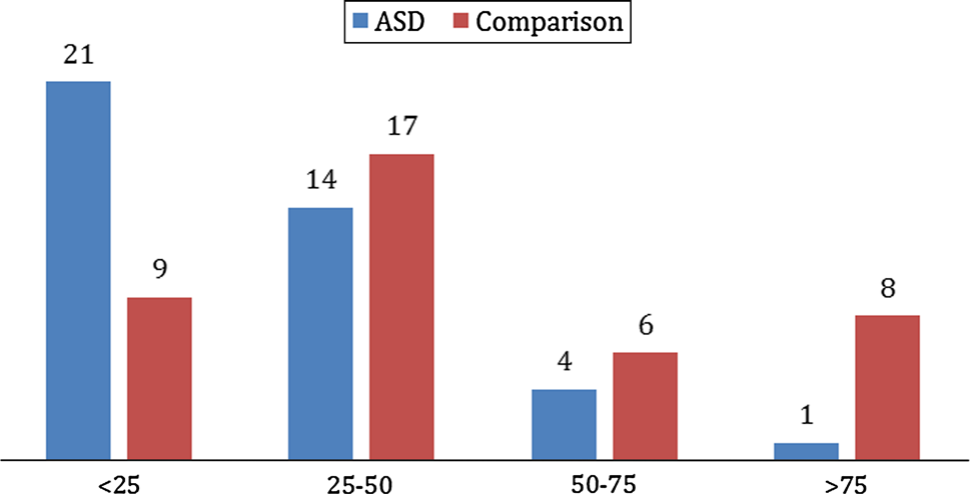
25(OH)D_3_ status among ASD and comparison groups (95 % CI, *p* = 0.003)

In the ASD group, 88 % were vitamin D deficient (Fig. 2). Among their siblings, parents, and the comparison group, the corresponding rates were 58, 58, and 65 % respectively (*p* < 0.001) (Table 1): for clarity and comparability of our results with several recently published studies of vitamin D levels in patients with autism we resolved to use the same reference ranges as (Holick et al. 2011) and we used values of nmol per litre (nmol/L).

**Table 1:** Vitamin D status: Chi squared test or table of percentages of participants with severe deficiency, deficiency, insufficiency and sufficiency of vitamin D (nmol/L) in the comparison group, ASD group and in siblings and parents of the individuals with ASD (Pearson Chi square = 26.730, *df* = 9, *p* = 0.002)

Vitamin D status (nmol/L)	Count % within groups	Groups				Total
		Comparison	ASD	Sibling	Parent	
Severe deficiency <25	Count	9	21	10	18	58
	%	22.5	52.5	16.1	23.4	25.5
Deficiency 25–50	Count	17	14	26	27	84
	%	42.5	35.0	41.9	35.1	38.4
Insufficiency 50–75	Count	6	4	17	13	40
	%	15.0	10.0	27.4	16.9	18.3
Sufficiency >75	Count	8	1	9	19	37
	%	20.0	2.5	14.5	24.7	16.9
Total	Count	40	40	62	77	219
	%	100	100	100	100	100

### Analysis of Season of Blood Sampling

Medians of vitamin D levels of individuals with ASD varied according to season of the year in which they were sampled: spring (n = 9) = 13.80 nmol/L, summer (n = 18) = 39.90 nmol/L and autumn (n = 12) = 22.95 nmol/L (*p* = 0.017) reflecting a similar trend as that found in the study of Faroese elder population (Dalgård et al. 2010). Therefore, the vitamin D data was adjusted for month of sampling in our analysis.

### Comparison of 25(OH)D_3_ Levels Across and Within Gender

The trend for males having lower vitamin D levels was observed in the ASD and sibling groups, although this gender difference was statistically significant only in the sibling group (*p* = 0.03). In the comparison and parent groups both males and females had comparable levels (Table 2).

**Table 2:** 25(OH)D_3_ gender differences in individual groups

GROUP (n)	Vitamin D level median (nmol/L)		*p* (CI 95 %)
	Male	Female	
ASD (40)	24.70	42.00	0.1 (5.6–31.1)
Comparison (40)	37.40	43.95	0.6 (12.7–23.5)
Siblings (62)	34.60	54.00	0.03 (1.2–27.3)
Parents (77)	44.90	44.45	0.9 (11.7–11.29)

Because of the variability in male:female ratio in all groups (ASD group ~3:1; sibling and parent groups ~1:1 and the comparison group ~2:1), a combined comparison of vitamin D levels was also carried out for ‘males only’ in all groups. There was a significant difference between the groups (*p* = 0.001). Males with ASD had significantly lower levels of 25(OH)D_3_ [median (IQR) = 24.7 (20.6) nmol/L] compared to their brothers [median (IQR) = 34.6 (25.2) nmol/L] (*p* = 0.004) and fathers [median (IQR) = 44.9 (49.8) nmol/L] (*p* < 0.001) and also to the healthy comparison males [median (IQR) = 37.4 (31.0) nmol/L] (*p* = 0.002).

### Comparison of 25(OH)D_3_ Levels in Individuals with ASD Recruited in 2002 and 2009

As there were only 2 females diagnosed in 2002 and one of them was ‘an outlier’ i.e. her 25(OH)D_3_ level was much higher than all other participants’ at 153 nmol/L, it was not meaningful to compare 25(OH)D_3_ levels across gender in this 2002 group. In the 2009 group there was no difference between males [median 24.9 nmol/L (IQR = 20.3)] and females [median 22.2 (IQR = 27.1)] (*p* = 0.37).

For comparison between 2002 and 2009 groups this outlier was removed—there was no difference between the 25(OH)D_3_ levels of the 2002 group [median (IQR) = 24.7 (28.80) nmol/L] and the 2009 group [median(IQR) = 23.6 (24.38) nmol/L] (95 % CI 9.10–11.40) (*p* = 0.965).

Among the 24 participants (22 males/2 females) recruited in 2002, 12 (50 %) had severely deficient, eight (30 %) deficient, three (13 %) insufficient, and one (=female) (4 %) sufficient levels of 25(OH)D_3_.

Among the 16 participants (9 males/7females) recruited in 2009, nine (56 %) had severely deficient, six (38 %) deficient, one (=female) (6 %) insufficient, and zero (0 %) sufficient levels of 25(OH)D_3_.

Although the 2002 and 2009 cohorts were diagnosed at two different time points, it should be noted that both groups were blood sampled for vitamin D analysis during the same period in 2008–2009.

### 25(OH)D_3_ Levels and Other Variables

There was no association between 25(OH)D_3_ levels and age, IQ, subcategories of ASD or ADOS score. When investigating ‘season of birth’ in the ASD group, those born in the spring season (March–May) had the lowest levels of 25(OH)D_3_ (median 13.8 nmol/L; *p* = 0.17) in agreement with previous literature (Grant and Soles 2009). Among the ASD group there were 13 (32.5 %) spring births (the comparison group was matched for season of birth therefore included the same number of spring births). There was no correlation between 25(OH)D_3_ levels and ADOS scores (*p* = 0.3). There was also no correlation between 25(OH)D_3_ levels and the diagnosis—Asperger syndrome (n = 18): 23.55 nmol/L (20.2); atypical autism (n = 11): 24.7 nmol/L (29.6); autism (n = 11): 30.3 (51.8) (*p* = 0.3).

## Discussion

This first-ever population-based study of vitamin D in ASD showed significantly lower levels in young adolescents/adults with ASD than in their siblings and parents, and also than healthy comparisons.

Our findings are consistent with those of several studies published since 2010, although some of these had methodological problems e.g., no comparison group or a problematic comparison group (Nseir et al. 2012; Kočovská et al. 2012a, b) (see “Appendix”).

The finding that 80 % of the comparison group (which can be considered representative of the general population in the Faroe Islands) were in the ‘deficient/insufficient’ range and 22.5 % in the ‘severely deficient’ range (<25 nmol/L–<10 ng/mL) that is indicative of the risk of osteomalacia/rickets (Holick 2007), adds to the mosaic of perceived global vitamin D deficiency in various regions of the world. It is of note that the levels of 25(OH)D_3_ found in ASD participants of the present study from the Faroe Islands are even lower than those in other regions (see “Appendix”). It might be that the severity of this region’s climate and its high Northern latitude, both diminishing the production of vitamin D by UVB rays, cannot be compensated for by a diet albeit rich in large oily fish.

The only other report on vitamin D levels in the Faroe Islands was the Faroese elder study (Dalgård et al. 2010) that found the median 25(OH)D_3_ concentration of 47.6 (29.8–64.8) nmol/L and a small seasonal variation in the 25(OH)D_3_ levels in Faroese elders, with a winter nadir of 42.6 nmol/L and a summer peak of 56.5 nmol/L (females 51.0 nmol/L; males 44.6 nmol/L). Our study found a similarly small, insignificant seasonal variation in our ASD group corresponding to the season of blood sampling.

Unlike in the Meguid (2010) and Gong (2014) studies our group with the most severe diagnosis of autism had the highest levels of vitamin D (30.30 nmol/L; *p* = 0.3). This could be partially explained by the fact that 9 out of 11 individuals with autism diagnoses were blood sampled during the summer holidays while for the two other diagnoses there was a greater variability in season of sampling.

The slight over-representation of spring births among the ASD group (32.5 %) and corresponding lowest level of vitamin D in this group (median 13.8 nmol/L; *p* = 0.17) is interesting and warrants follow up. A hypothetical explanation as to why ASD cases born in the spring had the lowest 25(OH)D_3_ status in adolescence could be that low maternal 25(OH)D_3_ levels led to epigenetic processes at the CYP24A1 gene. This gene encodes the 24-hydroxylase that degrades calcitriol and 25(OH)D_3_ is subject to epigenetic regulation (methylation) during neonatal life (Novakovic et al. 2009): epigenetic marking and silencing of the CYP24A1 gene could occur due to low maternal vitamin D levels preventing 25(OH)D_3_ from reaching a level that adequately supports CNS development. Further investigation of this potential mechanism may be of interest. To date the Faroese population generally has not been supplementing with vitamin D, even in pregnancy (apart from ordinary multivitamins with no more than 400 IU of cholecalciferol or ergoclaciferol per day), and the use of solaria in the Faroe Islands is also rare.

The trend towards lower levels of vitamin D in males in ASD and sibling groups presented another interesting finding. There are no acknowledged sun exposure differences between males and females among Faroese adolescents. Dalgård’s Faroese elder study also reported that female gender was associated with higher 25(OH)D_3_ levels (Dalgård et al. 2010). Previous research has demonstrated various gender differences in vitamin D metabolism (Spach and Hayes 2005; Orton et al. 2006; Novakovic et al. 2009; Feldman et al. 2011). Thus, future research is warranted, as it would shed light on this phenomenon.

Here we only aimed at cross-sectional comparison of vitamin D levels and we did not investigate mechanisms involved in the differences found. It can be expected that both diet and availability of UVB rays can influence vitamin D levels. We did not measure calcium, parathyroid hormone (PTH), or phosphate, which might reveal more about the nature of the deficiency either from inadequate sun exposure and/or inadequate dietary intake (Holick 2007). An individual’s underlying hormonal imbalance could also play a role (Cannell and Grant 2013; Holick 2007; Feldman et al. 2011). The apparatus regulating the hormone calcitriol (e.g. vitamin D receptors, vitamin D binding protein, associated enzymes) is all under genetic control and might exacerbate environmentally determined low vitamin D status or amplify its consequences to elevate ASD risk (Fu et al. 2009; Ahn et al. 2010; Hiraki et al. 2013). Thus future research is expected to shed light on a possible role of genetic factors. We intend to follow up and treat the diagnosed hypovitaminosis D among participants with ASD and try to obtain further information on the origin of low vitamin D status.

We have no evidence regarding the direction of causality as the blood samples of our ASD group were drawn when they were 15–24 years of age. The low vitamin D levels in the ASD group could be either a result of autism impacting on a family/child’s lifestyle and/or diet (indoor activities, selective eater, etc.) or the underlying biology of autism altering the metabolism of vitamin D in some way or vitamin D deficiency itself contributing to the pathogenesis of ASD. As all groups were exposed to low levels of sunlight, the very low 25(OH)D_3_ in the ASD group suggests some other underlying pathogenic mechanism may be involved.

Children with Williams syndrome very often have high 25(OH)D_3_ levels in early infancy due to a single gene mutation, which also involves abnormal vitamin D metabolism (Feldman et al. 2011; Stamm et al. 2001). It would be interesting to explore whether altered vitamin D metabolism could also be involved in the development of autism. One of the supportive features for causality is biological mechanism (Hennekens et al. 1987).

High ‘Body Mass Index’ (BMI) has been associated with vitamin D deficiency (Holick 2007). We did not have exact BMI values available for either of our comparison groups. In our ASD group, eight participants (20 %) were noted by diagnosing clinicians as overweight/obese. Thus, this factor itself does not offer an exhaustive explanation for our overall result.

An increasing body of research indicates that ASD may be associated with a variety of complex immune dysregulations, including autoimmunity, and may have a neuro-immunecomponent (Cannell and Grant 2013; Mostafa and AL-Ayadhi 2012; Gentile et al. 2013). Thus, future investigation utilizing a combination of genetic, epigenetic, and mechanistic studies of this complex interplay between autism, vitamin D deficiency, steroid metabolism, and autoimmunity will be desirable.

### Limitations

The main limitations of the present study were the modest size of our sample, a lack of data regarding BMI—only known in the some of the ASD group and lacking in all other control groups—and a lack of information about the participants´ habits with regards to indoor/outdoor activities or their vitamin D supplementation. Also there was an inconsistency of blood sampling across seasons among various groups. These research findings require replication with larger numbers. Because it would be challenging for any one centre to produce data on sufficient number of children with ASD, a multicentre international collaboration would be desirable in order to achieve more conclusive results.

## Conclusions

This first-ever population study of vitamin D levels in ASD showed significantly lower vitamin D levels in participants with ASD (aged 15–24 years) living in the Faroe Islands, as compared to their siblings, parents, and typically developing comparisons. A trend of lower levels in males compared to females in the ASD, comparison and sibling groups was also observed.

The findings could reflect the consequences of ASD per se impacting on a person’s lifestyle and diet or the underlying biology of ASD impacting in some way directly on the metabolism of vitamin D. Alternatively, the low vitamin D levels could be an indication of life-long vitamin D deficiency in ASD, and this hormone deficiency could, at least theoretically, have been involved in early aberrant development of the brain in these individuals, leading to the development of ASD.

## Appendix

See Table 3.

**Table 3:** Overview of studies of vitamin D levels in individuals with autism

Author (year)	Country: study participants (n)	ASD group		Comparison group		*p*
		nmol/L	ng/mL	nmol/L	ng/mL	
Humble et al. (2010)*	Sweden: adults (121)	31.5	12.6	–		–
Molloy et al. (2010)	USA: boys 4–8 years (89)	49.4	19.8	44.0	17.6	0.4
Meguid et al. (2010)	Egypt: children 2–8 years (112)	71.3	28.5	100.3	40.1	<0.001
Mostafa and AL-Ayadhi (2012)	Saudi Arabia: children 5–12 years (80)	46.3	18.5	82.5	33.0	<0.001
De SouzaTostes et al. (2012)	Brazil: children ±7 years (48)	66.2	26.5	101.3	40.5	<0.001
Gong et al. (2014)	China: children ±4 years (96)	49.8	19.9	56.5	22.6	0.002
Kočovská et al. (present study)	Faroe Islands: young adults 15–24 years (219)	24.8	9.9	37.6	15.0	0.002

* The Humble et al. (2010) study did not have a control group. For more details see (Kočovská et al. 2012a)

## References

[CR1] Adams JS, Hewison M (2010). Update in vitamin D. Journal of Clinical Endocrinology and Metabolism.

[CR2] Agborsangaya C, Toriola AT, Grankvist K, Surcel HM, Holl K, Parkkila S (2010). The effects of storage time, sampling season on the stability of serum 25-hydroxy vitamin D, androstenedione. Nutrition and Cancer.

[CR3] Ahn J, Yu K, Stolzenberg-Solomon R, Simon KC, McCullough ML, Gallicchio L (2010). Genome-wide association study of circulating vitamin D levels. Human Molecular Genetics.

[CR4] American Psychiatric Association (1994). Diagnostic and statistical manual of mental disorders.

[CR5] Cannell JJ, Grant WB (2013). What is the role of vitamin D in Autism?. Dermato-Endocrinology.

[CR6] Coleman M, Gillberg C (2012). The autisms.

[CR7] Dalgård C., Petersen, M. S., Schmedes, A.V., Brandslund, I., Weihe, P., & Grandjean, P. (2010). *British Journal of Nutrition, 104*, 914–918. 10.1017/S0007114510001509 PMC4413010 20441671

[CR8] De Souza Tostes MH, Polonini HC, Gattaz WF, Raposo NRB, Baptista EB (2012). Low serum levels of 25-hydroxyvitamin D (25-OHD) in children with autism. Trends in Psychiatry and Psychotherapy.

[CR9] Dealberto MJ (2011). Prevalence of autism according to maternal immigrant status and ethnic origin. Acta Psychiatrica Scandinavica.

[CR10] Ellefsen A, Kampmann H, Billstedt E, Gillberg IC, Gillberg C (2007). Autism in the Faroe Islands. An epidemiological study. Journal of Autism and Developmental Disorders.

[CR11] Eyles DW, Smith S, Kinobe R, Hewison M, McGrath JJ (2005). Distribution of the vitamin D receptor and 1α-hydroxylase in human brain. Journal of Chemical Neuroanatomy.

[CR12] Eyles DW, Burne TH, McGrath JJ (2013). Vitamin D, effects on brain development, adult brain function and the links between low levels of vitamin D and neuropsychiatric disease. Frontiers in Neuroendocrinology.

[CR13] Feldman D, Pike WJ, Adams JS (2011). Vitamin D.

[CR14] Fernell E, Barnevik-Olsson M, Bågenholm G, Gillberg C, Gustafsson S, Sääf M (2010). Serum levels of 25-hydroxyvitamin D in mothers of Swedish and of Somali origin who have children with and without autism. Acta Paediatrica.

[CR15] Fu L, Yun F, Oczak M, Wong BYL, Vieth R, Cole DEC (2009). Common genetic variants of the vitamin D binding protein (DBP) predict differences in response of serum 25-hydroxyvitamin D [25(OH)D] to vitamin D supplementation. Clinical Biochemistry.

[CR16] Garcion E, Wion-Barbot N, Montero-Menei CN, Berger F, Wion D (2002). New clues about vitamin D functions in the nervous system. Trends in Endocrinology and Metabolism.

[CR17] Gardener H, Spiegelman D, Buka SL (2009). Prenatal risk factors for autism: Comprehensive meta-analysis. The British Journal of Psychiatry.

[CR18] Gentile I, Zappulo E, Militerni R, Pascotto A, Borgia G (2013). Etiopathogenesis of autism spectrum disorders: Fitting the pieces of the puzzle together. Medical Hypotheses.

[CR101] Giarelli E, Wiggins LD, Rice CE, Levy SE, Kirby RS, Pinto-Martin J (2010). Sex differences in the evaluation and diagnosis of autism spectrum disorders among children. Disability and Health Journal.

[CR19] Gillberg C (2010). The ESSENCE in child psychiatry: Early symptomatic syndromes eliciting neurodevelopmental clinical examinations. Research in Developmental Disabilities.

[CR20] Gong ZL, Luo CM, Wang L, Shen L, Wei F, Tong RJ, Liu Y (2014). Serum 25-hydroxyvitamin D levels in Chinese children with autism spectrum disorders. NeuroReport.

[CR21] Grant WB, Soles CM (2009). Epidemiologic evidence supporting the role of maternal vitamin D deficiency as a risk factor for the development of infantile autism. Dermato-Endocrinology.

[CR22] Hayes CE, Nashold FE, Spach KM, Pedersen LB (2003). The immunological functions of the vitamin D endocrine system. Cellular and Molecular Biology (Noisy-le-grand).

[CR102] Heaney RP, Holick MF (2011). Why the IOM recommendations for vitamin D are deficient. Journal of Bone and Mineral Research.

[CR23] Hennekens CH, Buring JE, Mayrent SL (1987). Epidemiology in medicine.

[CR24] Hiraki LT, Major JM, Chen C, Cornelis MC, Hunter DJ, Rimm EB (2013). Exploring the genetic architecture of circulating 25-hydroxyvitamin D. Genetic Epidemiology.

[CR25] Holick MF (2007). Vitamin D deficiency. New England Journal of Medicine.

[CR26] Holick MF, Binkley NC, Bischoff-Ferrari HA, Gordon CM, Hanley DA, Heaney RP (2011). Evaluation, treatment, and prevention of vitamin D deficiency: An endocrine society clinical practice guideline. The Journal of Clinical Endocrinology and Metabolism.

[CR27] Humble MB, Gustafsson S, Bejerot S (2010). Low serum levels of 25-hydroxyvitamin D (25-OHD) among psychiatric out-patients in Sweden: Relations with season, age, ethnic origin and psychiatric diagnosis. The Journal of Steroid Biochemistry and Molecular Biology.

[CR103] Kočovská, E., Billstedt, E., Ellefsen, Á., Kampmann, H., Gillberg, I. C., Biskupstø, R., et al. (2013). Autism in the Faroe Islands: Diagnostic stability from childhood to early adult life. *The Scientific World Journal, 2013*, 7. 10.1155/2013/592371. PMC3586480 23476144

[CR29] Kočovská E, Biskupstø R, Gillberg IC, Ellefsen Á, Kampmann H, Stórá T (2012). The rising prevalence of autism: A prospective longitudinal study in the Faroe Islands. Journal of Autism and Developmental Disorders.

[CR28] Kočovská E, Fernell E, Billstedt E, Minnis H, Gillberg C (2012). Vitamin D and autism: Clinical review. Research in Developmental Disabilities.

[CR30] Kolevzon A, Gross R, Reichenberg A (2007). Prenatal and perinatal risk factors for Autism. Archives of Pediatrics and Adolescent Medicine.

[CR100] Kopp S, Kelly K, Gillberg C (2010). Girls with social and/or attention deficits: A descriptive study of 100 clinic attenders. Journal of Attention Disorders.

[CR31] Lord C, Rutter M, Goode S, Heemsbergen J, Jordan H, Mawhood L (1989). Autism diagnostic observation schedule: A standardized observation of communicative and social behavior. Journal of Autism and Developmental Disorders.

[CR32] Meguid NA, Hashish AF, Anwar M, Sidhom G (2010). Reduced serum levels of 25-hydroxy and 1, 25-dihydroxy vitamin D in Egyptian children with autism. Journal of Alternative and Complementary Medicine.

[CR33] Molloy CA, Kalkwarf HJ, Manning-Courtney P, Mills JL, Hediger ML (2010). Plasma 25(OH)D concentration in children with autism spectrum disorder. Developmental Medicine and Child Neurology.

[CR34] Mostafa GA, AL-Ayadhi LY (2012). Reduced serum concentrations of 25-hydroxy vitamin D in children with autism: Relation to autoimmunity. Journal of Neuroinflammation.

[CR35] Munger KL, Levin LI, Massa J, Horst R, Orban T, Ascherio A (2012). Preclinical serum 25-hydroxyvitamin D levels and risk of type 1 diabetes in a cohort of US military personnel. American Journal of Epidemiology.

[CR36] Novakovic B, Sibson M, Ng HK, Manualpillai U, Rakyan V, Down T (2009). Placenta-specific methylation of the vitamin D 24-hydroxylase gene. The Journal of biological chemistry.

[CR37] Nseir W, Mograbi J, Abu-Rahmeh Z, Mahamid M, Abu-Elheja O, Shalata A (2012). The association between vitamin D levels and recurrent group A streptococcal tonsillopharyngitis in adults. International Journal of Infectious Diseases.

[CR38] Orton S-M, Herrera BM, Yee IM, Valdar W, Ramagopalan SV, Sadovnick AD (2006). Sex ratio of multiple sclerosis in Canada: A longitudinal study. Lancet Neurology.

[CR104] Priemel, M., von Domarus, C., Klatte, T. O., Kessler, S., Schlie, J., Meier, S., et al. (2010). Bone mineralization defects and vitamin D deficiency: Histomorphometric analysis of iliac crest biopsies and circulating 25-hydroxyvitamin D in 675 patients. *Journal of Bone and Mineral Research, 25*, 305–312. 10.1359/jbmr.090728 19594303

[CR39] Ramagopalan SV, Heger A, Berlanga AJ, Maugeri NJ, Lincoln LR, Burrell A (2010). A ChIP-seq-defined genome-wide map of vitamin D receptor binding: Associations with disease and evolution. Genome Research.

[CR40] Schöttker B, Jansen EH, Haug U, Schomburg L, Köhrle J, Brenner H (2012). Standardization of misleading immunoassay based 25-hydroxy-vitamin D levels with liquid chromatography tandem-mass spectrometry in a large cohort study. PLoS ONE.

[CR41] Shea RL, Berg JD (2013). Measuring 25-hydroxy vitamin D in haemolysed whole blood samples. Annals of Clinical Biochemistry.

[CR42] Spach KM, Hayes CE (2005). Vitamin D_3_ confers protection from autoimmune encephalomyelitis only in female mice. The Journal of Immunology.

[CR43] Stamm C, Friehs I, Ho SY, Moran AM, Jonas RA, del Nido PJ (2001). Congenital supravalvar aortic stenosis: A simple lesion?. European Journal of Cardio-Thoracic Surgery.

[CR44] Wechsler D (1981). Wechsler adult intelligence scale-revised, 1981.

[CR45] Wechsler D (1992). Wechsler Intelligence Scale for Children.

[CR46] Wing L, Leekam SR, Libby SJ, Gould J, Larcombe M (2002). The diagnostic interview for social and communication disorders: Background, inter-rater reliability and clinical use. Journal of Child Psychology and Psychiatry.

[CR47] World Health Organization (1993). The ICD-10 classification of mental and behavioural disorders. Diagnostic criteria for research.

